# Evaluation of a free-breathing myocardial T_1_ mapping using magnetization-prepared slice interleaved spoiled gradient echo imaging in patient

**DOI:** 10.1186/1532-429X-17-S1-Q130

**Published:** 2015-02-03

**Authors:** Jihye Jang, Sébastien Roujol, Tamer A Basha, Shingo Kato, Sophie Berg, Warren J Manning, Reza Nezafat

**Affiliations:** 1 Department of Medicine, Beth Israel Deaconess Medical Center and Harvard Medical School, Boston, MA, USA; 2 Computer Aided Medical Procedures, Technische Universität München, Munich, Germany; 3 Department of Radiology, Beth Israel Deaconess Medical Center and Harvard Medical School, Boston, MA, USA

## Background

Quantitative myocardial T_1_ mapping shows promise for assessment of various cardiomyopathies. Most available sequences are generally acquired within a breath-hold using a balanced SSFP (bSSFP) imaging readout. However, the signal obtained from a bSSFP imaging readout is T_2_ dependent, sensitive to magnetization transfer and has increased susceptibility to the B_0_ field inhomogeneity leading to regional variations in T_1_ estimates (1). Recently, we developed a novel T_1_ mapping sequence for free-breathing, multi-slice, myocardial T_1_ mapping using a slice-interleaved T_1_ sequence with spoiled gradient echo (GRE) imaging readout. However, the feasibility of this sequence in patient has not yet been studied. In this study, we sought to demonstrate the feasibility of this sequence in patients and its ability to detect abnormal T_1_ times.

## Methods

Sixteen patients referred to clinical CMR for assessment of cardiomyopathy (54±21y, 13 m) and eleven control healthy adult subjects (35±21y, 4 m) were recruited for this study. Subjects were scanned on a 1.5 T Philips scanner. Native T_1_ mapping was performed using a free-breathing slice interleaved acquisition which enables simultaneous acquisition of 5 slices using multiple inversion recovery (IR) experiments (2). All the 5 slices are acquired once in each IR experiment, which enables the sampling of the undisturbed T_1_ recovery curve. Each T_1_-weighted image was acquired using an ECG-triggered acquisition with a GRE imaging readout (TR/TE/α=4.3/2.1ms/10˚, FOV=280×272 mm^2^, voxel size=2×2 mm^2^, slice thickness=8 mm, 5 slices, number of phase-encoding lines=43, linear ordering, 10 linear ramp-up pulses, SENSE factor=2.5, half Fourier=0.75, bandwidth=382Hz/pixel). Prospective slice tracking was combined with retrospective image registration to correct for respiratory motion. Myocardial native T_1_ values were evaluated using a 16 myocardial segment model for all healthy subjects and patients.

## Results

**Figure 1:**
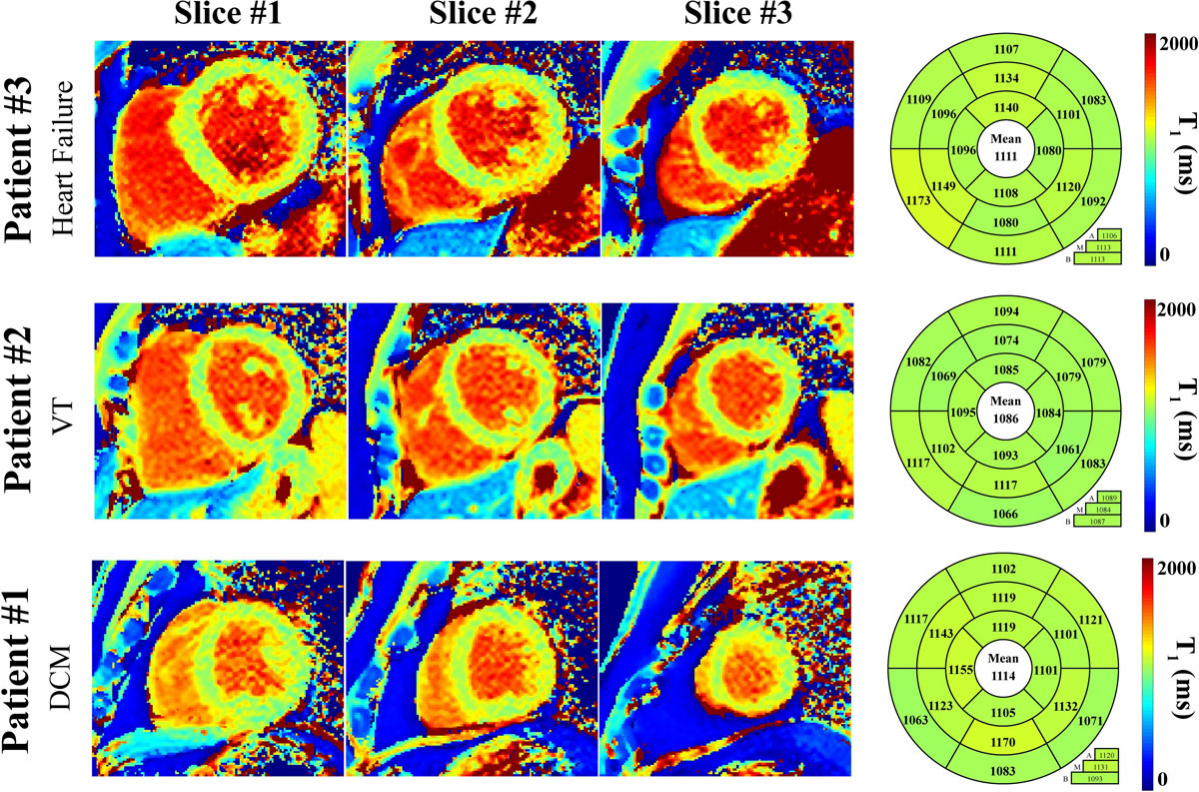
Example of native T_1_ maps obtained in three patients using the proposed sequence. Each map was evaluated using a 16 myocardial segment model using the three mid-ventricular slices. These patients were referred to CMR with the following indications: Patient #1-dilated cardiomyopathy (DCM), patient #2-ventricular tachycardia (VT) and patient #3-heart failure. Homogeneous and consistent T_1_ times were obtained over all myocardial segments. Native T_1_ times obtained in these patients was similar to the average native T_1_ time of the healthy subject control group (1094±24ms).

**Figure 2:**
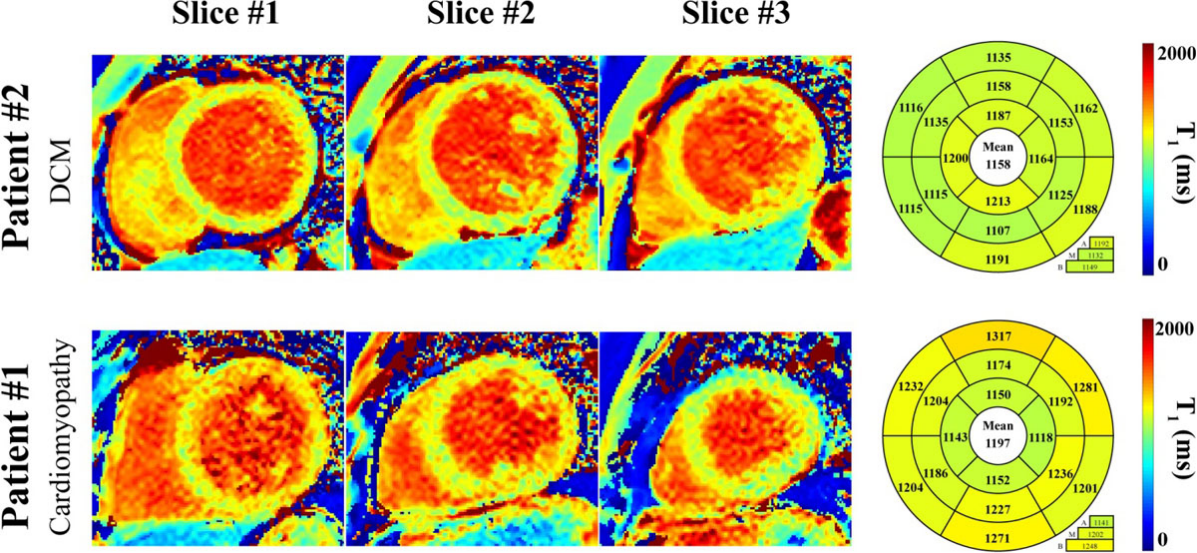
Example of native T_1_ maps obtained in two patients using the proposed sequence. Each map was quantified using a 16 myocardial segment model using the three mid-ventricular slices. These patients were referred to CMR with the following indications: Patient #1-assessment of cardiomyopathy and patient #2-dilated cardiomyopathy (DCM). Elevated native T_1_ times were observed in these patients when compared to the healthy subject control group (1094±24ms).

Native T_1_ times in the healthy subject control group were 1094±24ms. Figure 1 shows an example of native T_1_ maps obtained in three patients where native T_1_ times (1114ms, 1086ms, and 1111ms) were in the same range as in the healthy subject control group. Homogeneous and consistent T_1_ times were obtained over the whole myocardium in these patients and the visual quality of T_1_ map was excellent. Figure 2 shows an example of native T_1_ maps obtained in two patients where elevated T_1_ times can be observed (1197ms and 1158ms). Elevated T_1_ times were consistent across multiple myocardial segments.

## Conclusions

Free-breathing myocardial T_1_ mapping using magnetization-prepared slice interleaved spoiled gradient echo imaging is feasible in patients and provides excellent T_1_ map quality which enables the detection of altered native T_1_ times in the presence of specific cardiomyopathies.
